# Beyond monetary benefits of restoring sight in Vietnam: Evaluating well-being gains from cataract surgery

**DOI:** 10.1371/journal.pone.0192774

**Published:** 2018-02-12

**Authors:** Simon Feeny, Alberto Posso, Lachlan McDonald, Truong Thi Kim Chuyen, Son Thanh Tung

**Affiliations:** 1 International Development and Trade Research Group, RMIT University, Victoria, Australia; 2 Ho Chi Minh City University of Social Sciences and Humanities, Ho Chi Minh City, Vietnam; Sun Yat-Sen University Zhongshan Ophthalmic Center, CHINA

## Abstract

A more holistic understanding of the benefits of sight-restoring cataract surgery requires a focus that goes beyond income and employment, to include a wider array of well-being measures. The objective of this study is to examine the monetary and non-monetary benefits of cataract surgery on both patients as well as their caregivers in Vietnam. Participants were randomly recruited from a Ho-Chi-Minh City Hospital. A total of 82 cataract patients and 83 caregivers participated in the survey conducted for this study. Paired *t*-tests, Wilcoxon Signed Rank tests, and regression analysis are used to detect any statistically significant differences in various measures of well-being for patients and caregivers before and after surgery. There are statistically significant improvements in monetary and non-monetary measures of well-being for both patients and caregivers approximately three months after undergoing cataract surgery, compared with baseline assessments collected prior to surgery. Non-monetary measures of well-being include self-assessments of overall health, mental health, hope, self-efficacy, happiness and life satisfaction. For patients, the benefits included statistically significant improvements in earnings, mobility, self-care, the ability to undertake daily activities, self-assessed health and mental health, life satisfaction, hope, and self-efficacy (*p*<0.01). For caregivers, attendance at work improved alongside overall health, mental health, hope, self-efficacy, happiness and life satisfaction, three months post-surgery (*p*<0.01). Restoring sight has positive impacts for those suffering from cataracts and their caregivers. Sometimes the benefits are almost equal in their magnitude. The study has also demonstrated that many of these impacts are non-monetary in nature. It is clear that estimates of the rate of return to restoring sight that focus only on financial gains will underestimate the true returns to society of restoring sight from cataract surgeries.

## Introduction

Since economic reforms beginning in 1986, Vietnam has experienced rapid economic growth and made great progress towards the Millennium Development Goals. The government has also implemented a nationwide social health insurance scheme known as Vietnam Social Security (VSS). However, not all members of the population have benefitted from these structural transformations. These inequities are reflected in eye health. As is the case around the world, untreated cataracts are the largest cause of blindness and low vision in Vietnam [1]. Despite being included in the VSS scheme, cataract surgery is not universally accessible. Economic and geographic barriers disadvantage poor and vulnerable groups, including women [2,3].

Vision loss robs people of their productivity, health and happiness. Poverty and blindness are tightly linked through a cycle in which poverty increases the risk of visual impairment and visual impairment causes poverty [4]. Moreover, there is a link between vision loss and co-morbidities (e.g. injuries sustained from falls) [5]. People with vision loss are also more susceptible to mental illness [6]. Caregivers (usually women and girls) are also economically disadvantaged as the time they spend caring comes at the expense of being otherwise productive [7].

International studies confirm that successful cataract surgeries lead to important economic and quality of life improvements for patients and that these benefits can accrue quickly [8]. However, such studies have tended to focus on improved labour market outcomes [9,10]. As such, when caregivers are factored into the analysis it is often to highlight foregone earning opportunities [11]. However, the large literature on surgical interventions and well-being suggests that the benefits of restoring sight should extend beyond the additional earning capacity of individuals and include a raft of other benefits to both patients and caregivers [11].

This study makes a number of contributions to understanding the broader benefits of cataract surgery. It looks at changes in measures of well-being by studying patients both before and after surgical interventions. It incorporates a wider variety of well-being measures than existing studies that focus on either economic or psychological measures, but not both. It also focuses on patients and caregivers in poor communities in a developing country where formal support networks are relatively limited and, as such, people are more reliant on family for support. Hence the role the caregiver plays is potentially large.

## Methods

Written consent was obtained from all participants. Visually impaired participants were read a participant information statement in the presence of their caregiver and were asked to sign the consent form in the presence of the surveyor and caregiver. Written forms with the signatures of all participants are safely stored at in the Ho-Chi-Minh City University of Social Sciences. Ethics approval was obtained from the Human Research Ethics Committee of RMIT University and the Ho-Chi-Minh City Ophthalmologic Hospital Ethics Committee. Both ethics committees reviewed and approved the consent forms.

Sample size was based on the following calculations. Using demographic data available from the Vietnam census and Household Living Standards Surveys, coupled with a 99% confidence level and a 5% 2-sided significance level, the minimum sample size was estimated to be 65 patients and caregivers, respectively. To err on the side of caution we targeted a sample size equal to 20% above the required minimum. The overall sample is comprised of 82 patients and 83 caregivers, surveyed both before and three months after cataract surgery.

The initial period of data collection began in late-August to mid-October 2016. The Ho-Chi-Minh City Ophthalmologic Hospital was approached to assist with the recruitment of participants. Potential participants were asked whether they needed help with their daily tasks due to their visual impairment. To be eligible for the research project the person needed to respond ‘yes’. The sample therefore captures patients who were either blind or visually impaired.

Separate surveys for patients and caregivers were prepared. The questionnaires were based on a similar survey used in Vietnam [10] while the sampling techniques outlined in Grosh and Glewwe [12] were used. The patient survey captures the severity of visual impairment with questions that measure the extent of functional vision using a 4-point Likert scale, identifying whether they have difficulties with activities such as reading newspapers, recognising faces, reading text on the TV, or, more generally, in any way in their daily life.

Both patient and carer questionnaires collected information on the economic activities of patients and caregivers with questions about their monthly incomes and how much time they spend on various activities. These questionnaires are augmented with measures of physical and mental health using international survey instruments developed by the World Health Organization (WHO) for use in the World Mental Health Survey Initiative. Mental health questions from the Rand Corporation’s Rand 36-item Health Survey were also included to capture whether individuals feel (i) nervous, (ii) calm and peaceful, (iii) happy, or (iv) depressed. The survey also examines levels of hope using an index created by Snyder *et al*. [13]. It is based on the responses to six questions and can take values between 0 and 36. Self-efficacy is measured using Schwarzer and Jerusalem’s self-efficacy scale [14]. It is based on the response to 10 questions and can take values between 10 and 40.

### Demographics of the sample

The sample is comprised of poorer members of the Vietnamese population that were eligible for subsidised treatment from the charity campus of the Ho-Chi-Minh City Ophthalmologic Hospital. The sample was also restricted to willing participants living in and around Ho-Chi-Minh City. There was a near- equal gender split for patients while caregivers were marginally more likely to be female. Most patients in the sample (76% (62/82)) were aged over 50; while caregivers tended to be younger (57% (47/83) were aged under 50). Virtually all patients, pre-surgery, were cared for by a family member and over 80% (65/82) co-habit.

#### Statistical analysis

Preliminary analysis uses paired *t*-tests and Wilcoxon Signed Rank tests (for ordinal variables) to identify statistically significant differences in well-being measures pre- and post-surgery. We test for the robustness of our findings with multivariate regressions using a fixed effects specification that controls for unobserved time-invariant characteristics that influence the variables of interest.

## Results

### The success of surgery: Before and after comparisons

Cataract surgery resulted in an improvement in functional vision for nearly all patients, often to a point of having no difficulty seeing. Prior to surgery, 88% (72/82) of patients reported that their present vision gives them ‘great difficulty’ or ‘very great difficulty’ in their daily life. The same percentage reported ‘no difficulty’ following cataract surgery.

Wilcoxon Signed Rank tests confirmed statistically significant improvements in being able to (i) read text in the newspaper (*p*<0.01); (ii) recognise the faces of people they meet (*p*<0.01); (iii) see the prices of goods when shopping (*p*<0.01); (iv) see uneven ground when walking (*p*<0.01); (v) see when doing needlework and handicrafts (*p*<0.01); (vi) read text on the TV (*p*<0.01); and (vii) see to carry out a preferred hobby (*p*<0.01). Approximately three quarters of the surgeries conducted in the sample were freely provided. Most of the remaining surgeries cost VND 2.7 million (approximately US$120).

### Changes in employment and work

About three quarters of patients had an occupation of some sort (pre-surgery) but 40% (33/82) reported doing a different occupation before they became visually impaired. The vast majority reported earning more money from their prior occupation–implying that their income had fallen since the onset of their visual impairment. At the time the pre-surgery survey was conducted, 48% (40/82) of patients reported that they were working. This increased to 58% (48/83) approximately three months after surgery. The improvement is statistically significant at the one per cent level. The change in the work status of caregivers is not statistically significant (see Table 1).

**Table 1:** Changes in patient and carer income and employment.

Indicator	Before Surgery	After surgery	Change
Patient monthly income (million dong)	1.6	2.4	0.8**
Patient work status (per cent)	48 (40/83)	58 (48/83)	10
Caregiver monthly income (million dong)	3.9	4.1	0.23
Caregiver work status (per cent)	86 (71/83)	81 (67/83)	-5

** denotes *p*<0.05.

Calculations based on 83 observations. Fractions resulting in percentages are in brackets.

Significance levels for income and work status are obtained from pairwise *t*-tests and Wilcoxon Signed Rank tests, respectively. Source: The authors.

Survey data also indicated that incomes can increase quickly when sight is restored. Pairwise *t*-tests reveal that three months after surgery, average incomes of patients had increased by approximately 54% (1.6 to 2.4 million dong) (*p*<0.05). Similarly, 36% (30/83) of caregivers indicated that their income increased since their dependent had the surgery (3.9 to 4.1 million dong), although this change is found to be statistically insignificant. Table 1 summarizes these findings.

### Changes in autonomy

These changes in income for patients and caregivers tell an important part of the story surrounding the benefits of health interventions. However, it is also important to highlight the sense of independence, autonomy and self-realisation that these individuals receive when they command higher income.

Using a 3-point Likert scale (low, moderate, high), patients were asked to rate their level of difficulty moving around, taking care of themselves and performing daily activities, before and after surgery. On average, 30% (25/82) of patients reported some level of difficulty with the three indicators prior to surgery. This proportion declined to around 10% (8/82) following surgery. Wilcoxon Signed Rank tests reveal that this drop of approximately 20 percentage points is found to be statistically significant across all indicators. Table 2 summarises the results.

**Table 2:** Changes in patient measures of autonomy, per cent.

Indicator	Before Surgery	After surgery	Change
Mobility (per cent of patients reporting moderate or high difficulty in walking)	34 (28/83)	13 (11/83)	22**
Self-care (per cent of patients reporting moderate or high difficulty in washing or dressing themselves)	25 (21/83)	5 (4/83)	20***
Performing activities (per cent of patients reporting moderate or high difficulty in working, studying, doing housework or leisure activities)	39 (32/83)	7 (6/83)	32***

** denote 5 per cent levels of significance

*** denote 1 per cent levels of significance

Calculations based on 83 observations. Fractions resulting in percentages are in brackets. Significance levels are obtained from Wilcoxon Signed Rank tests. Source: The authors.

### Changes in health

Prior to surgery, 46% (38/82) of patients rated their overall health as ‘poor’. After surgery only 6% (5/82) rated their health as ‘poor’, while 44% (36/82) rated it as ‘very good’ or ‘excellent’. Interestingly, improvements in self-assessed health were also high for caregivers. Remarkably, the proportion of caregivers reporting their health as ‘very good’ or ‘excellent’ increased substantially, from 13% (11/83) to 45% (37/83), after their dependent had surgery. Wilcoxon Signed Rank tests reveal that both improvements are statistically significant at the 1% level.

### Changes in self-assessed mental health

**Fig 1:**
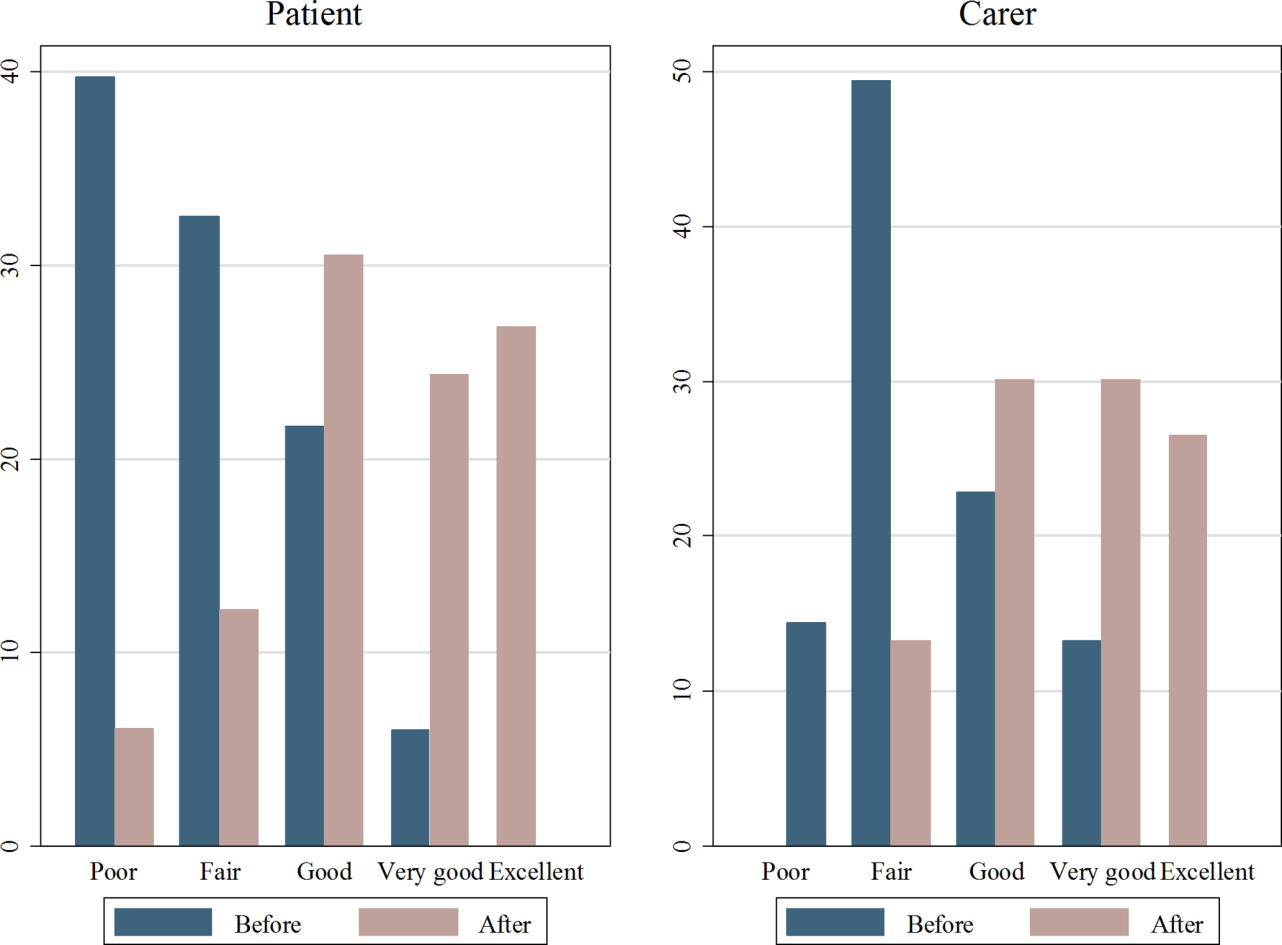
Patient and carer self-assessed mental health before and after surgery. Source: The authors.

Self-assessed mental health is found to significantly increase for both patients and caregivers three months after the surgery (see Fig 1 below). Patients reporting that their mental health was ‘very good’ or ‘excellent’ increased from 6% (5/82) to 51% (42/82). For caregivers, the figure rose from 13% (11/82) to 57% (47/83). According to Wilcoxon Signed Rank tests, both of these improvements are statistically significant at the 1% level.

**Fig 2:**
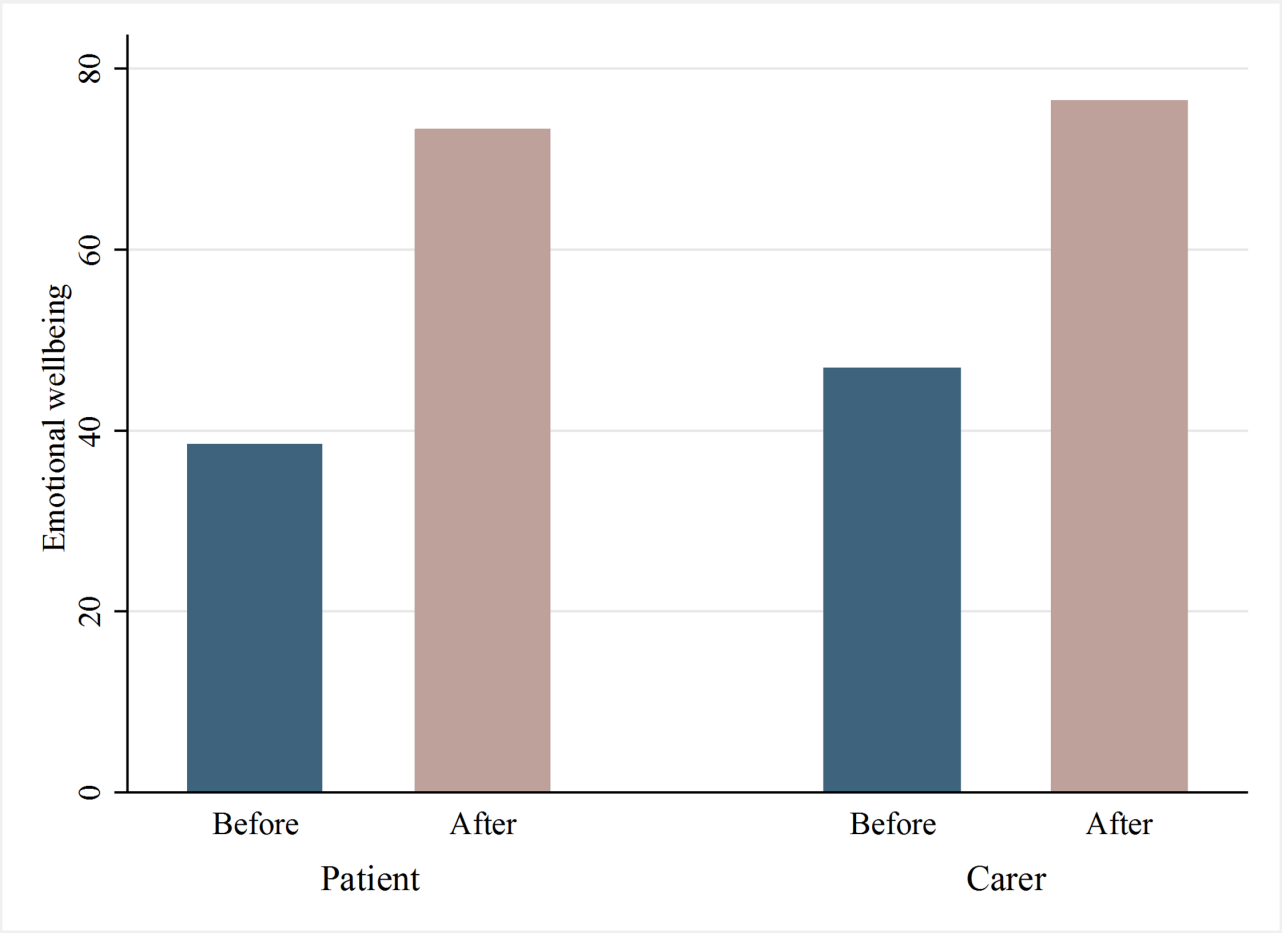
Emotional well-being, before and after surgery. Source: The authors.

Other measures of mental health were also captured by the survey. Respondents were asked questions about how often they have felt the following during the past four weeks: nervous; calm and peaceful; downhearted and sad; and happy. Wilcoxon Signed Rank tests reveal that there were statistically significant improvements in all of these measures for both patients and caregivers at the 1% level. An index of mental health was constructed which combines these different measures. It takes values from 0 (extremely low mental health) to 100 (very high mental health). Wilcoxon Signed Rank tests reveal that emotional well-being improved for both caregivers and patients following surgery. Prior to surgery, the average patient exhibited a score of 39 for their mental health, while the corresponding figure for carers was 47. After surgery, patients’ score increased to 73 (*p*<0.01), while carers increased to 76 (*p*<0.01) (Fig 2). Responses to questions about anxiety and depression were used to test the robustness of these results. Patients and carers were asked to rate their level of anxiety/depression using a 3-point Likert scale (not anxious or depressed, moderately anxious or depressed, highly anxious or depressed). Prior to surgery, 88% (72/82) and 77% (64/83) of patients and caregivers indicated that they suffered from moderate or high levels of anxiety or depression, respectively. Following the surgeries, the corresponding figures fell to 24% (20/82) (*p*<0.01) and 1% (1/83) (*p*<0.01), respectively.

**Fig 3:**
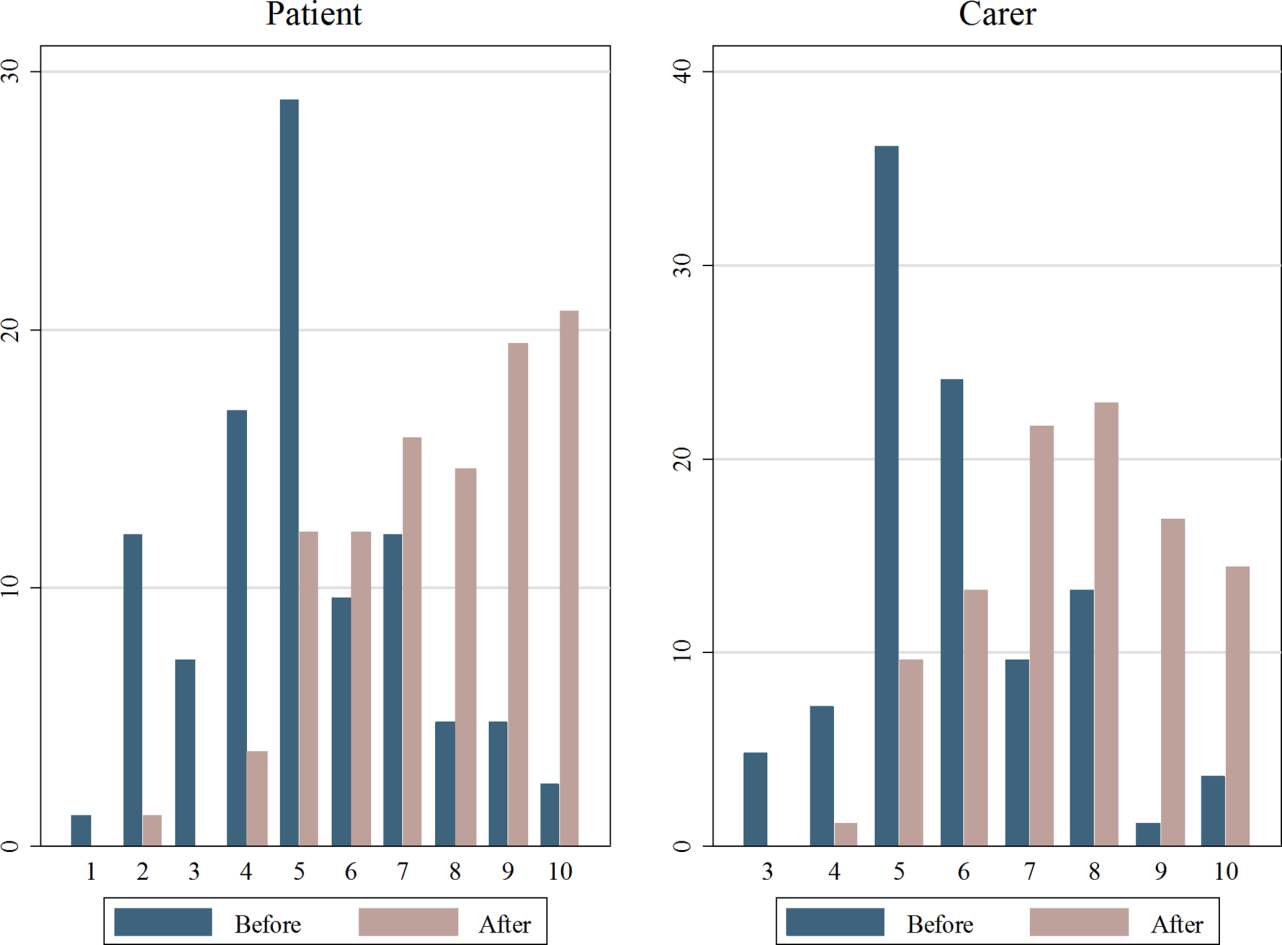
Patient and carer self-assessed life-satisfaction before and after. Source: The authors.

Given that levels of self-assessed overall health and mental health both increased after surgery, it is not surprising that overall life-satisfaction also rose. Life-satisfaction is measured using a question that asked respondents to rate, on a scale of 1–10, how happy they are (with one being not happy and 10 being very happy). Fig 3 presents the findings. On average, the level of happiness for patients is 2.5 points higher after cataract surgery (7.6 versus 5.1) (*p*<0.01) and 1.7 points higher for caregivers (7.6 versus 5.9) (*p*<0.01).

**Fig 4:**
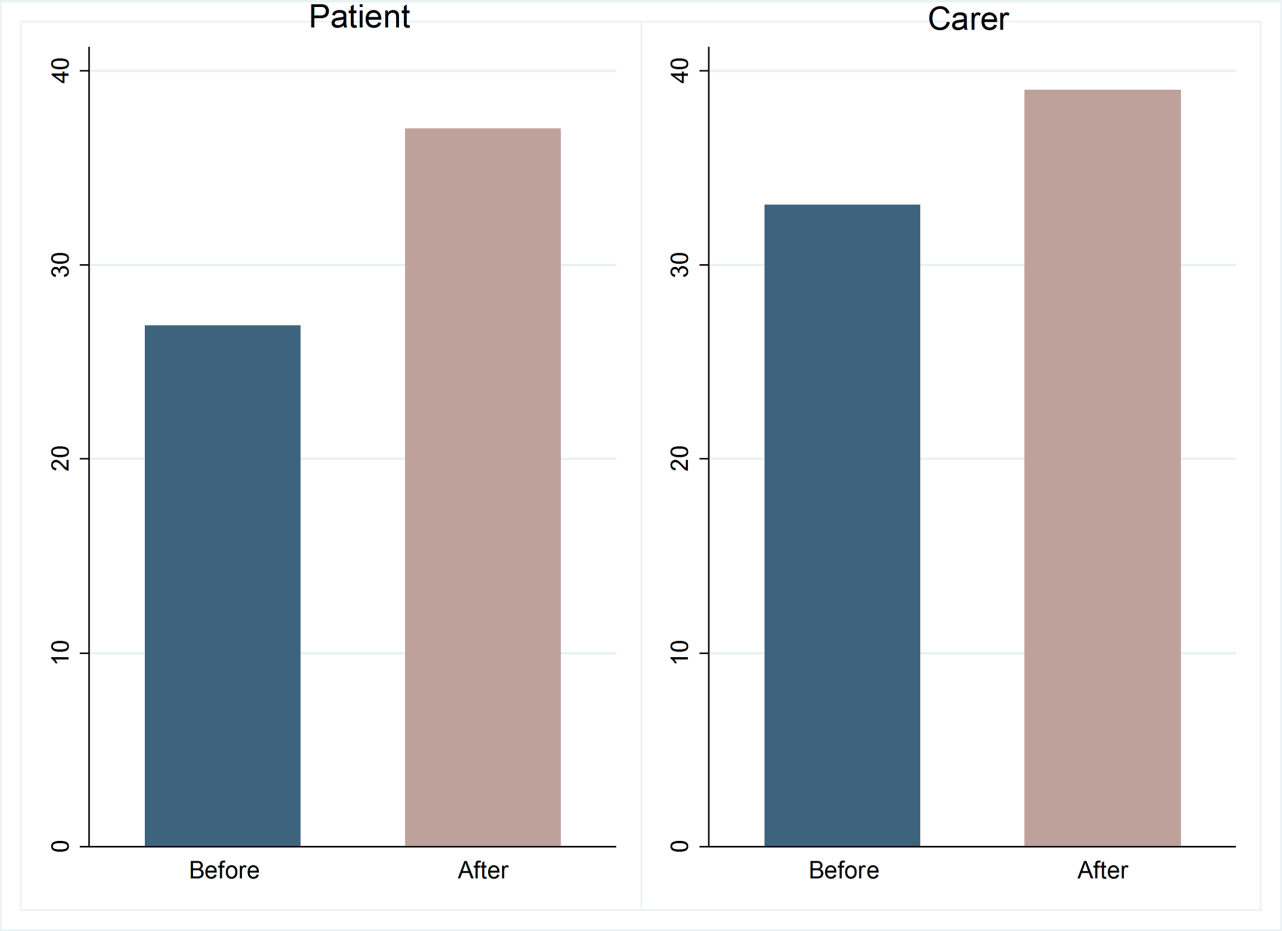
Patient and carer average hope, before and after surgery. Source: The authors.

Wilcoxon Signed Rank tests confirm that hope and self-efficacy exhibit statistically significant improvements for both patients and caregivers following cataract surgery. Hope improves by more than 10 points for patients (37.5 versus 27.2) (*p*<0.01) and almost 6 points for caregivers on average (39.0 versus 33.1) (*p*<0.01). Hope is comprised of two core elements: agency and pathway. Agency relates to having goal-directed energy while pathway relates to the planning to accomplish goals. For both patients and caregivers, the pathway component increases by a little more than agency following surgery. As above, the analysis shows that improvements are statistically significant at the 1% level. Fig 4 summarises these findings for patients and caregivers, respectively.

Similar findings are reported for self-efficacy. Self-efficacy improved by between 4 (*p*<0.01) and 5 (*p*<0.01) points on average for patients and caregivers respectively following surgery, demonstrating that the restoration of sight has important impacts on their beliefs of their capabilities to undertake tasks and cope with different situations.

### Regression analyses

In order to test whether the patterns in the data that are obtained above are robust to the inclusion of other key variables of interest, we employ fixed-effects regression techniques. Exploiting the fact that we have data for the same individuals across two periods we can fit regressions that control for time-invariant omitted variables that could potentially influence the variables of interest. These variables include factors such as ability, gender and educational attainment [15]. The results in Table 3 confirm the findings above. That is, after controlling for other variables and unobserved factors, all outcomes exhibit statistically significant improvements after surgery.

**Table 3:** Regression analysis, patient and caregiver fixed effects.

	(1)	(2)	(3)	(4)	(5)
Dependent variable:	Income (000’s)	Well-being	Emotional Well-being	Health	Mental health
**Panel A: Patients**					
Post-surgery	9.26**	7.22*	6.40*	3.85**	2.41**
	[2.18]	[1.77]	[1.74]	[2.15]	[2.32]
Observations	165	165	165	96	96
R-squared	0.09	0.70	0.75		
Chi-squared p-value				0	0
**Panel B: caregivers**					
Post-surgery	229.8**	31.4***	29.8***	2.72***	3.74***
	[2.03]	[9.11]	[10.2]	[3.73]	[3.69]
Observations	166	166	166	68	88
R-squared	0.05	0.51	0.56		
Chi-squared p-value				0	0

* denote 10 per cent levels of significance

** denote 5 per cent levels of significance

*** denote 1 per cent levels of significance

Robust t-statistics in brackets. All regressions include individual-level fixed effects. The patient regressions include a control that asks respondents if they’re satisfied with their vision. The carer regressions include a control that asks respondents if they live with the patient. The health and mental health regressions use dummy variables equal to 1 if the respondent self-classifies as having ‘good’, ‘very good, or excellent heath/mental health.

## Discussion

This study focuses on Vietnam, a middle-income country with comparatively good healthcare, but with large unmet demand for cataract surgeries. Cataract surgery is already considered to be among the most cost-effective interventions in health care [16,17]. It is also known to deliver large economic benefits and improve the quality of life of patients. Studies of the impacts of cataract surgery have tended to focus on the financial improvements–increased income, wealth and employment–and/or have centred on the patient themselves.

This study confirms previous work that the benefits of restoring sight are substantial. It extends the existing evidence base to show that cataract surgery has broader non-monetary impacts on patients as well as their caregivers. These include improved physical and mental health as well as a number of psychosocial measures of well-being. We find statistically significant improvements in all measures of well-being for both patients and, importantly, their caregivers. Indeed, caregivers are often subject to low levels of income and psychological well-being akin to the visually impaired.

Our findings are naturally subject to a number of important limitations. First, survey findings emanate from a small sample of just 82 cataract patients and 83 caregivers. Secondly, the sample is not nationally representative, with most participants being poorer members of Vietnam’s population since they were eligible for subsidised treatment from the charity campus of the Ho-Chi-Minh City Ophthalmologic Hospital. The sample was also restricted to willing participants living in and around Ho-Chi-Minh City. Third, the study examines impacts on a number of measures of well-being. However, for the survey these were selected on an *ad hoc* basis rather than being drawn from an established underlying conceptual framework or theory of change. Further, all outcomes but particularly psychosocial measures such as hope and self-efficacy are prone to large measurement error. Finally, the study assesses outcomes at just one point of time: three months after surgery.

Future work could attribute shadow prices to these effects in order to include them in a measure such as a Social Return on Investment Analysis. Doubtless, if appropriately valued, these true benefits would permit benefit-to-cost ratios to dwarf current estimates. Indeed, it is rare in international development to identify such large social impacts from a proven intervention. This is particularly true when cataract surgery is fully or partly subsidised for the poor in developing countries. Further studies should also be based on larger samples of participants which would allow for sub-group analysis to detect differences in benefits for different groups within the population. For example, the impacts of restoring sight might differ between the poor and the non-poor, men and women, the young versus the old, the rural versus the urban, and the employed versus unemployed. Outcomes could also be compared across sufferers of different eye diseases, for example cataract versus glaucoma patients.

Other research in the region may also highlight implications of cataract interventions in ways that are more pertinent to other developing nations. Indeed, Vietnam enjoys a comparatively strong public health sector, which is not necessarily the case in some of its neighbouring countries. Replicating this work in nations with more significant health-resource constraints is likely to highlight how economic development interacts with both the benefits and costs of eye disease.

## Conclusions

This study finds that cataract surgery considerably improves the well-being of patients and their caregivers. Improvements are observed in monetary dimensions of well-being, including employment, work and income, and non-monetary dimensions such as autonomy, self-assessed health, mental health, life satisfaction, hope, and self-efficacy. With untreated cataract remaining the world’s largest source of avoidable blindness these impacts show that money spent on expanding cataract surgical capacity is a sound, and much-needed, social investment. Future research should examine whether outcomes over a longer post-surgery period (such as six months or a year) persist or increase.

## Supporting information

S1 File Patient.dta. Anonymized patient data for use in STATA.
(DTA)

S2 File Patient.xls. Anonymized patient data for use in MS Excel.
(XLS)

S3 File Carer.dta. Anonymized caregiver data for use in STATA.
(DTA)

S4 File Carer.xls. Anonymized caregiver data for use in MS Excel.
(XLS)
